# Intranasal 15d-PGJ2 inhibits the growth of rat lactotroph pituitary neuroendocrine tumors by inducing PPARγ-dependent apoptotic and autophagic cell death

**DOI:** 10.3389/fnins.2023.1109675

**Published:** 2023-05-12

**Authors:** Zongyang Li, Lei Chen, Di Zhang, Xianjian Huang, Jihu Yang, Weiping Li, Chuanfang Wang, Xiangbao Meng, Guodong Huang

**Affiliations:** Department of Neurosurgery, Shenzhen Second People’s Hospital, School of Pharmaceutial Science, Health Science Center, Shenzhen University, Shenzhen, China

**Keywords:** PPARγ, pituitary neuroendocrine tumors, apoptosis, autophagy, 15d-PGJ2

## Abstract

PPARγ agonists have been reported to induce cell death in pituitary neuroendocrine tumor (PitNET) cell cultures. However, the therapeutic effects of PPARγ agonists *in vivo* remain unclear. In the present study, we found that intranasal 15d-PGJ2, an endogenous PPARγ agonist, resulted in growth suppression of Fischer 344 rat lactotroph PitNETs induced by subcutaneous implantation with a mini-osmotic pump containing estradiol. Intranasal 15d-PGJ2 reduced the volume and weight of the pituitary gland and the level of serum prolactin (PRL) in rat lactotroph PitNETs. 15d-PGJ2 treatment attenuated pathological changes and significantly decreased the ratio of PRL/pituitary-specific transcription factor 1 (Pit-1) and estrogen receptor α (ERα)/Pit-1 double-positive cells. Moreover, 15d-PGJ2 treatment induced apoptosis in the pituitary gland characterized by an increased ratio of TUNEL-positive cells, cleavage of caspase-3, and elevated activity of caspase-3. 15d-PGJ2 treatment decreased the levels of cytokines, including TNF-α, IL-1β, and IL-6. Furthermore, 15d-PGJ2 treatment markedly increased the protein expression of PPARγ and blocked autophagic flux, as evidenced by the accumulation of LC3-II and SQSTM1/p62 and the decrease in LAMP-1 expression. Importantly, all these effects mediated by 15d-PGJ2 were abolished by cotreatment with the PPARγ antagonist GW9662. In conclusion, intranasal 15d-PGJ2 suppressed the growth of rat lactotroph PitNETs by inducing PPARγ-dependent apoptotic and autophagic cell death. Therefore, 15d-PGJ2 may be a potential new drug for lactotroph PitNETs.

## 1. Introduction

The most common pituitary neuroendocrine tumors (PitNETs) are of lactotroph, somatotroph, thyrotroph, corticotroph, and gonadotroph lineages (Neou et al., 2020). The detailed cellular and molecular mechanisms of PitNET tumorigenesis, development and pathogenesis are still poorly understood. Lactotroph PitNETs, which express and/or secrete prolactin (PRL), can cause various symptoms, such as hyperprolactinemia, galactorrhea, amenorrhea, infertility, headache, and visual defects (Srirangam Nadhamuni and Korbonits, 2020). In the clinic, lactotroph PitNETs are usually effectively controlled with dopamine D2 receptor agonists, including bromocriptine and cabergoline, or transsphenoidal surgery (Zhang et al., 2022). However, some lactotroph PitNET patients are resistant to dopamine agonists or suffer from cerebrospinal rhinorrhea or hypopituitarism after surgery (Pivonello et al., 2021). Therefore, there is a need to better understand the pathogenesis and to develop novel therapeutic strategies for lactotroph PitNETs.

Peroxisome proliferator-activated receptor γ (PPARγ) is a type II nuclear receptor involved in regulating various genes, including glucose and lipid metabolism (Montaigne et al., 2021). PPARγ has been implicated in a wide range of diseases, including type 2 diabetes, neurodegenerative disorders, and PitNETs (Soccio et al., 2014; Cai et al., 2018; Jamwal et al., 2021). Activation of PPARγ by 15d-PGJ2 promotes apoptosis in a large number of cancer cell types (Wasinger et al., 2014; Colin et al., 2018; Mikulčić et al., 2021). Interestingly, rosiglitazone reduced the levels of growth hormone (GH) and insulin-like growth factor 1 (IGF-1) in patients with acromegaly and promoted apoptosis and autophagy in primary somatotroph adenoma and GH3 cells (Zhang et al., 2021). These findings suggest that activation of PPARγ by 15d-PGJ2 may offer a novel therapeutic strategy for PitNETs.

An increasing number of studies have demonstrated that many drugs are involved in the activation of autophagic cell death in PitNETs (Leng et al., 2017; Tae et al., 2018), highlighting the importance of autophagy in tumor therapy. Macroautophagy (hereafter referred to as autophagy) is a cellular process involving the self-degradation and recycling of cellular components (Tae et al., 2018). Autophagy is vital for cell homeostasis and functions (Levy et al., 2017). Complete autophagy includes initiation, elongation, autophagosome formation, maturation, autophagosome-lysosome fusion, and degradation of content in autophagolysosomes. The role of autophagy in PitNETs is complex (Tulipano and Giustina, 2021). It may act as a tumor survival factor as well as a tumor suppressor in a context-dependent manner (Weckman et al., 2014). Autophagy can be triggered by a variety of chemotherapy drugs. The dopamine D2 receptor agonist bromocriptine induces autophagy-dependent cell death in pituitary adenomas (Geng et al., 2017). The dopamine D2 receptor agonist cabergoline induced pituitary lactotroph PitNET cell death via blockade of autophagic flux, leading to autophagic cell death (Tang et al., 2019). The combination of cabergoline and chloroquine may increase clinical effectiveness in the treatment of human pituitary adenomas by blocking normal autophagic cycles and inducing apoptosis (Lin et al., 2017).

We previously found that PPARγ agonists could regulate autophagy (Li et al., 2022). The PPARγ agonist 15d-PGJ2 has been reported to exhibit anticancer activity by inducing apoptotic or autophagic cell death pathways (Bie et al., 2018). 15d-PGJ2 exerts a proapoptotic effect via ROS-dependent activation of transcription factor EB (TFEB) and subsequent regulation of autophagy (Yang et al., 2022). However, the effect of 15d-PGJ2 on lactotroph PitNETs *in vivo* and the underlying mechanisms are not fully understood.

In the present study, we found that intranasal 15d-PGJ2 inhibited the growth of rat lactotroph PitNETs by inducing PPARγ-dependent apoptotic and autophagic cell death.

## 2. Materials and methods

### 2.1. Materials

Estradiol (E2, HY-B0141, purity = 99.99%), 15d-PGJ2 (HY-108568, purity ≥ 97.0%), GW9662 (HY-16578, purity = 99.87%), and SBE-β-CD (purity ≥ 98.0%; HY-17031) were supplied by MedChemExpress (Shanghai, China). Bovine serum albumin (BSA), Triton X-100, isoflurane and paraformaldehyde were purchased from Sigma–Aldrich (MO, United States). Anti-pituitary-specific transcription factor 1 (Pit-1, ab273048), anti-NF-κB p65 (ab16502), anti-microtubule-associated protein 1 light chain 3B (LC3B, ab192890), anti-sequestosome 1 (SQSTM1/p62, ab109012), anti-lysosome-associated membrane protein-1 (LAMP-1, ab24170), and anti-beta tubulin (ab6046) primary antibodies and Alexa Fluor^®^ 647-conjugated goat anti-mouse IgG H&L (ab150115), Alexa Fluor^®^ 488-conjugated goat anti-rabbit IgG H&L (ab150077), HRP-conjugated goat anti-rabbit IgG H&L (ab6721), and HRP-conjugated goat anti-mouse IgG H&L (ab6789) secondary antibodies were all purchased from Abcam (CA, United States). A rat PRL enzyme-linked immunosorbent assay (ELISA) kit (ERA50RBX10) and mouse PRL monoclonal antibody (MA1-10597), mouse estrogen receptor α (ERα) monoclonal antibody (MA1-310), mouse PPARγ monoclonal antibody (419300), and rabbit caspase-3 polyclonal antibody (PA5-78921) were obtained from Invitrogen (CA, United States). The bicinchoninic acid (BCA) kit and enhanced chemiluminescence (ECL) kit were purchased from Pierce Biotechnology (IL, United States).

### 2.2. Animal model of lactotroph PitNETs

All animal experimental procedures were approved by the Ethics Committee of Shenzhen Second People’s Hospital and conducted in accordance with the National Institutes of Health Guide for the Care and Use of Laboratory Animals. The animal model of lactotroph PitNETs was established by subcutaneous administration of E2. Ten-week-old female Fischer 344 rats were obtained from Vital River Laboratories (Beijing, China) and kept under specific pathogen-free conditions, with a 12 h light/12 h dark cycle and were fed *ad libitum*. Fifty rats were ovariectomized (OVX) to exhaust endogenous estrogen. After a week of recovery, the OVX rats were assigned randomly to 5 groups (*n* = 10) and implanted subcutaneously with Alzet osmotic pumps (Model 2006, DURECT corporation, CA, United States) containing E2 (1, 2, 4, 8, and 16 mg). Ten age-matched rats were sham-operated and implanted with pumps containing only vehicle (1% DMSO + 99% saline containing 20% SBE-β-CD). The body weights of the rats were monitored weekly. Six weeks after pump implantation, the rats were anesthetized with isoflurane. Blood samples were then collected from the femoral arteries of the rats. The serum was isolated by centrifugation and stored at −80°C for PRL ELISA. The pituitary glands of rats were immediately removed and weighed after decapitation.

### 2.3. Drug treatments

For dose selection of 15d-PGJ2, fifty OVX + E2 rats were assigned randomly to 5 groups (*n* = 10) after the establishment of lactotroph PitNETs was validated via magnetic resonance imaging (MRI) at the fourth week of E2 (8 mg) treatment: vehicle (100 μL/day), 15d-PGJ2 (1 μg/100 μL/day), 15d-PGJ2 (2 μg/100 μL/day), 15d-PGJ2 (4 μg/100 μL/day) and GW9662 (1 μg/100 μL/day). 20 sham rats were assigned randomly to 2 groups: vehicle and 15d-PGJ2 (4 μg/100 μL/day). 15d-PGJ2 and GW9662 were dissolved in 1% (v/v) DMSO/99% (v/v) saline containing 20% SBE-β-CD. The rats were administered 15d-PGJ2, GW9662 or vehicle intranasally for 14 days. The rats were kept supine, and a droplet of 10 μL of the appropriate treatment was administered using a pipette into each nostril. Then, the rats were kept supine for 30 s and allowed to walk for 1 min. The same cycle was repeated 10 times. At day 14 after drug administration, the pituitary glands of rats were scanned by MRI. Ten age-matched rats were sham operated, implanted with pumps containing vehicle, and administered vehicle. The body weights of the rats were monitored weekly. The rats were anesthetized with isoflurane. Blood samples were then collected from the femoral arteries of the rats. The serum was isolated by centrifugation and stored at −80°C for PRL ELISA. The pituitary glands of rats were immediately removed and weighed after decapitation.

In the following experiments, sixty OVX + E2 rats were randomly assigned into 3 groups after the lactotroph PitNETs were validated via MRI at the fourth week of E2 (8 mg) treatment: vehicle, 15d-PGJ2 (4 μg/100 μL/day), and GW9662 (1 μg/100 μL/day) + 15d-PGJ2 (4 μg/100 μL/day). Each group consisted of 20 rats. Twenty age-matched rats were used as sham controls.

The body weights of the rats were monitored weekly. After completion of drug treatments, 10 rats were randomly selected from each group and anesthetized with isoflurane. Blood samples were then collected from the femoral arteries of the rats. The serum was isolated by centrifugation and stored at −80°C for PRL ELISA. Fourteen rats were randomly selected from each group, anesthetized with isoflurane, and then perfused transcardially with 60 mL of ice-cold saline. The pituitary glands were immediately removed, weighed and immediately frozen for ELISA (*n* = 8 per group) and western blotting (*n* = 6 per group). The other rats (*n* = 6 per group) were anesthetized with isoflurane and then perfused with 60 mL of ice-cold saline, followed by 60 mL of 4% paraformaldehyde. The pituitary glands (*n* = 3 per group) were randomly collected for hematoxylin and eosin (HE) staining, TUNEL staining, and immunofluorescence. The other pituitary glands (*n* = 3 per group) were dissected for transmission electron microscopy (TEM) analysis.

### 2.4. MRI

Pituitary MRI scanning was performed on a 7.0 Tesla vertical bore Bruker Biospec 70/30 scanner (BrukerBioSpin MRI GmbH, Rheinstetten, Germany). The parameters used in coronal MRI scans were optimized for gray/white matter contrast: T2-weighted, 3D fast spin–echo sequence, with TR = 4,000 ms, echo train length = 8, TEeff (Echo Time) = 36 ms, field-of-view (FOV) = 30 × 30 × 15 mm, matrix size = 256 × 256 × 30, voxel size = 0.1171875 × 0.1171875 × 0.5 mm, and total imaging time = 14.5 min. For axial and sagittal MRI scans, TR = 2,500 ms, echo train length = 8, TEeff (Echo Time) = 36 ms, field-of-view (FOV) = 30 × 30 × 10 mm, matrix size = 384 × 384 × 20, voxel size = 0.078125 × 0.075125 × 0.5 mm, and total imaging time = 14 min. The volumes of pituitary glands were calculated with 3D Slicer software.

### 2.5. Elisa

The levels of serum PRL (*n* = 10 per group) were detected by ELISA according to the manufacturer’s instructions. The pituitary glands (*n* = 8 per group) were homogenized in ice-cold RIPA buffer containing phosphatase inhibitor and protease inhibitor cocktail (Roche Diagnostics, Basel, Switzerland). The homogenates were centrifuged at 4°C and 20,000 rpm for 10 min, and the resulting supernatants were pooled for the analysis of caspase-3 activity and the levels of inflammatory cytokines, including TNF-α, IL-1β, and IL-6, according to the manufacturer’s instructions.

### 2.6. He staining

The pituitary glands (*n* = 3 per group) were fixed in 4% paraformaldehyde, embedded in paraffin, and sectioned at 5 μm thickness. The sections were dewaxed in xylene, rehydrated in ethanol, stained with hematoxylin and eosin, and then scanned using a Pannoramic MIDI scanner (3DHISTECH, Budapest, Hungary). Images were obtained by observers who were blinded to the experimental groups.

### 2.7. TUNEL staining

The pituitary glands (*n* = 3 per group) were fixed in 4% paraformaldehyde, embedded in paraffin, and sectioned at 5 μm thickness. The sections were dewaxed in xylene and rehydrated in ethanol. The sections were subjected to 750 W microwave irradiation in citrate buffer (0.1 M, pH 6.0) for 1 min. After being washed with PBS, the sections were incubated with Tris-HCl (0.1 M, pH 7.5) containing 1% BSA for 30 min and then incubated with TUNEL reaction mixture at 37°C in a humidified atmosphere in the dark for 60 min using an *in situ* Cell Death Detection Kit (Cat. No. 11684795910, Roche, Mannheim, Germany). The nuclei were stained with DAPI. The sections were scanned using the Pannoramic MIDI scanner. Images were obtained by observers who were blinded to the experimental groups. The number of TUNEL-positive cells was counted from four randomly chosen fields, and the ratio of TUNEL-positive cells to total cells was calculated.

### 2.8. Immunofluorescence

The expression of PRL, ERα, PPARγ and Pit-1 in pituitary glands was measured by immunofluorescence assay. The pituitary glands (*n* = 3 per group) were fixed in 4% paraformaldehyde, embedded in paraffin, and sectioned into 5 μm pieces. The sections were dewaxed in xylene and rehydrated in ethanol. The sections were subjected to 750 W microwave irradiation in citrate buffer (0.1 M, pH 6.0) for 20 min. The sections were treated with 0.3% H_2_O_2_ in methanol at room temperature for 20 min to block endogenous peroxidase. The sections were incubated in 1% BSA containing 0.1% Triton X-100 in PBS at room temperature for 1 h and then coincubated with rabbit polyclonal anti-Pit-1 antibody and mouse monoclonal anti-ERα antibody, mouse monoclonal anti-PRL antibody or mouse monoclonal anti-PPARγ antibody at 4°C overnight. After being washed with PBS, the sections were incubated with Alexa Fluor^®^ 488-conjugated goat anti-rabbit IgG H&L and Alexa Fluor^®^ 647-conjugated goat anti-mouse IgG H&L at room temperature for 1 h. Cover slips were mounted in Gel Mount (Vectashield, CA, United States). The sections were scanned using the Pannoramic MIDI scanner. Images were obtained by observers who were blinded to the experimental groups. The number of PRL-immunoreactive cells, ERα-immunoreactive cells and Pit-1-immunoreactive cells were counted from four randomly chosen fields. The ratio of PRL-immunoreactive cells and ERα-immunoreactive cells to Pit-1-immunoreactive cells was calculated.

### 2.9. Tem

The pituitary glands (*n* = 3 per group) were fixed with 2.5% glutaraldehyde overnight and postfixed with 1% osmium tetroxide (pH 7.4) at room temperature for 2 h. The tissues were dehydrated in a series of graded acetone concentrations and then infiltrated and embedded in PolyBed (Polysciences). The tissues were then polymerized at 60°C for 48 h and sectioned at 60 nm thickness. The sections were stained with a 2% aqueous solution of uranyl acetate for 30 min and poststained with Venable’s lead citrate; then, images were captured with a transmission electron microscope (JEOL, Tokyo, Japan).

### 2.10. Western blotting

Pituitary glands (*n* = 6 per group) were obtained and homogenized in cold RIPA buffer containing a protease inhibitor cocktail, phosphatase inhibitor cocktail, and phenylmethanesulfonylfluoride (Roche Diagnostics, Basel, Switzerland) and then centrifuged at 4°C and 10,000 rpm for 10 min. The supernatants were collected, and the total protein concentration was measured using a BCA kit. Equal amounts of proteins were separated with 10% or 12% sodium dodecyl sulfate polyacrylamide gels and electrotransferred to polyvinylidene fluoride membranes (Bio-Rad, CA, United States). The membranes were blocked in 5% nonfat milk powder in Tris-buffered saline containing 0.1% Tween-20 (TBST) for 1 h. After incubating at 4°C overnight with anti-PPARγ, anti-LC3B, anti-SQSTM1/p62, anti-LAMP-1, anti-caspase-3 and anti-beta tubulin antibodies, the membranes were incubated with goat anti-rabbit IgG H&L (HRP) antibody or goat anti-mouse IgG H&L (HRP) antibody at room temperature for 1 h. The protein bands were visualized by a ChemiDoc Touch Imaging System (Bio-Rad, CA, United States) using ECL kits. The intensities of the protein bands were quantified by densitometry using Molecular Imager Image Lab software (Bio-Rad, CA, United States). All protein band densities were normalized to beta tubulin.

### 2.11. Statistical analysis

Data are presented as the means ± SDs. Statistical significance was assessed using IBM SPSS Statistics version 20 (SPSS Inc., IL, United States). Comparisons among three or more groups were conducted using one-way analysis of variance (ANOVA) followed by Tukey’s *post hoc* test. A *p* value less than 0.05 was considered statistically significant.

## 3. Results

### 3.1. Intranasal 15d-PGJ2 reduced tumor weight and serum PRL in E2-treated OVX rats

**Figure 1:**
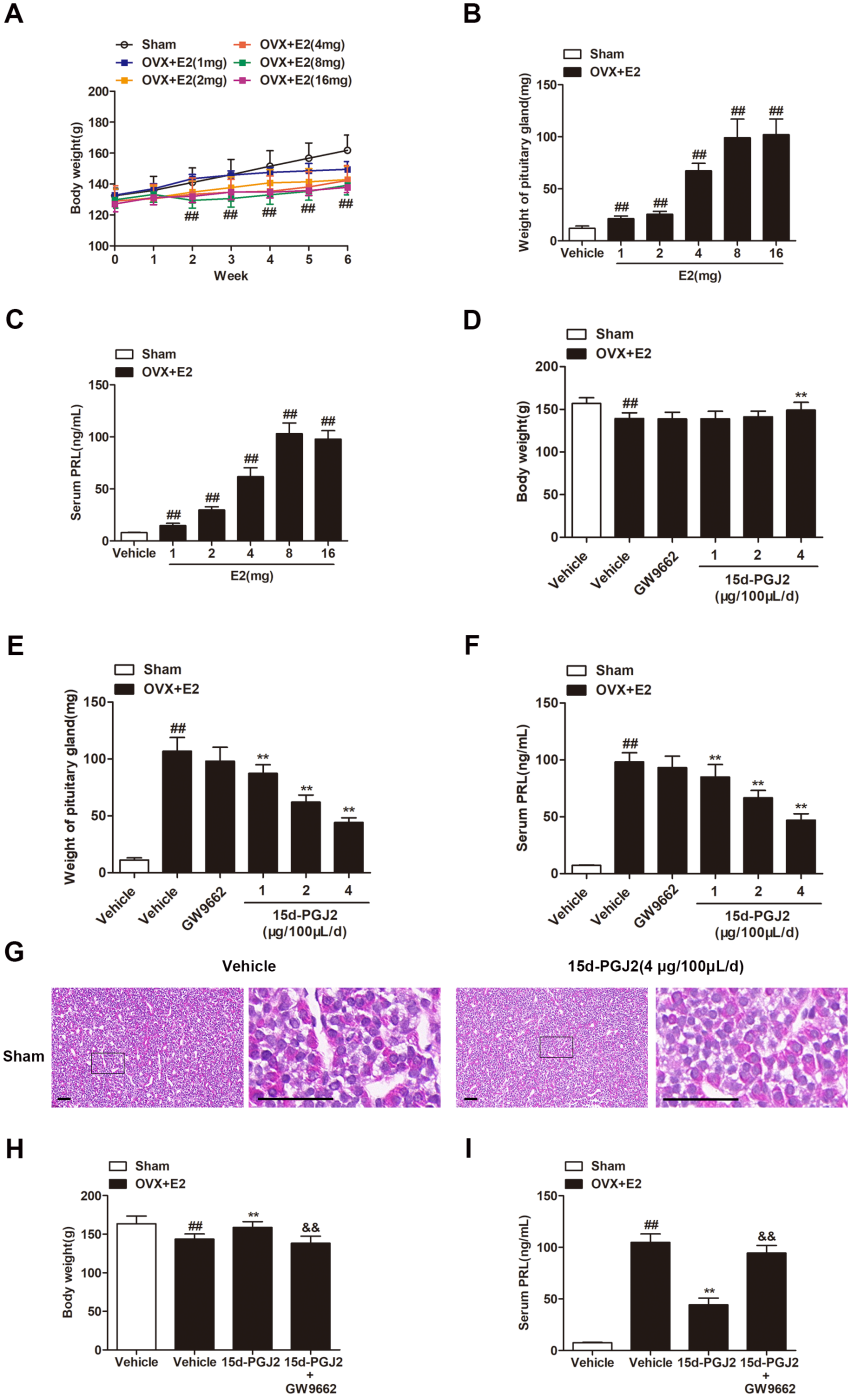
Intranasal 15d-PGJ2 reduced the tumor weight and serum PRL in E2-treated OVX rats. **(A)** The effects of E2 on the body weight of OVX rats (*n* = 10 per group). **(B)** The effects of E2 on the weight of the pituitary gland of OVX rats (*n* = 10 per group). **(C)** The effects of E2 on serum PRL in OVX rats (*n* = 10 per group). **(D)** The effects of 15d-PGJ2 on the body weight of E2-treated OVX rats (*n* = 10 per group). **(E)** The effects of 15d-PGJ2 on the pituitary gland weight of E2-treated OVX rats (*n* = 10 per group). **(F)** The effects of 15d-PGJ2 on serum PRL in E2-treated OVX rats (*n* = 10 per group). **(G)** Sham rats were treated with vehicle or 15d-PGJ2 (4 μg/100 μL/day) for 14 days. The pathological changes in the anterior pituitary glands of rats were detected by HE staining. Scale bar = 50 μm. **(H)** 15d-PGJ2 treatment increased the body weight of E2-treated OVX rats in a PPARγ-dependent manner (*n* = 20 per group). **(I)** 15d-PGJ2 treatment decreased serum PRL in E2-treated OVX rats in a PPARγ-dependent manner (*n* = 10 per group). Data are expressed as the means ± SDs. Statistical analysis was performed by one-way ANOVA followed by Tukey’s *post hoc* tests. ##, *p* < 0.01 versus Sham; **, *p* < 0.01 versus vehicle-treated OVX + E2 rats; &&, *p* < 0.01 versus 15d-PGJ2-treated OVX + E2 rats.

In this study, an animal model of lactotroph PitNETs was established by subcutaneous administration of E2. Treatment with E2 for 6 weeks significantly decreased the body weight of OVX Fischer 344 rats (*p* < 0.01, Figure 1A), whereas E2 substantially increased the weight of the pituitary glands of OVX rats in a dose-dependent manner (*p* < 0.01, Figure 1B). The volume of the pituitary gland was approximately 9-fold higher in E2-treated OVX rats than in sham rats. The ELISA results showed that E2-treated OVX rats displayed a marked increase in PRL levels compared to sham rats (*p* < 0.01, Figure 1C). The mean serum PRL levels in E2-treated OVX rats reached 100 ng/mL at a dose of 8 mg E2, while the mean PRL level in sham rats was 8 ng/mL. Therefore, the treatment with E2 (8 mg) for 4 weeks was selected for further experiments. Moreover, 15d-PGJ2 treatment significantly increased the body weight at a concentration of 4 μg/100 μL/day (*p* < 0.01, Figure 1D). Treatment with 15d-PGJ2 (1, 2, 4 μg/100 μL/day) for 14 days markedly decreased the pituitary gland weight and serum PRL level of E2-treated OVX rats in a dose-dependent manner (*p* < 0.01, Figures 1E,F). Sham rats were treated with vehicle or 15d-PGJ2 (4 μg/100 μL/day) for 14 days. There was no significant pathological change in the anterior pituitary glands of sham rats after treatment with 15d-PGJ2 (Figure 1G). Therefore, the treatment with 15d-PGJ2 (4 μg/100 μL/day) for 14 days was selected for further experiments. Conversely, cotreatment with 15d-PGJ2 (4 μg/100 μL/day) and GW9662 (1 μg/100 μL/day) abolished the 15d-PGJ2-mediated increase in body weight and the 15d-PGJ2-mediated decreases in the pituitary gland weight and serum PRL level of E2-treated OVX rats (*p* < 0.01, Figures 1H,I).

### 3.2. Intranasal 15d-PGJ2 reduced the tumor weight and serum PRL in E2-treated OVX rats in a PPARγ-dependent manner

**Figure 2:**
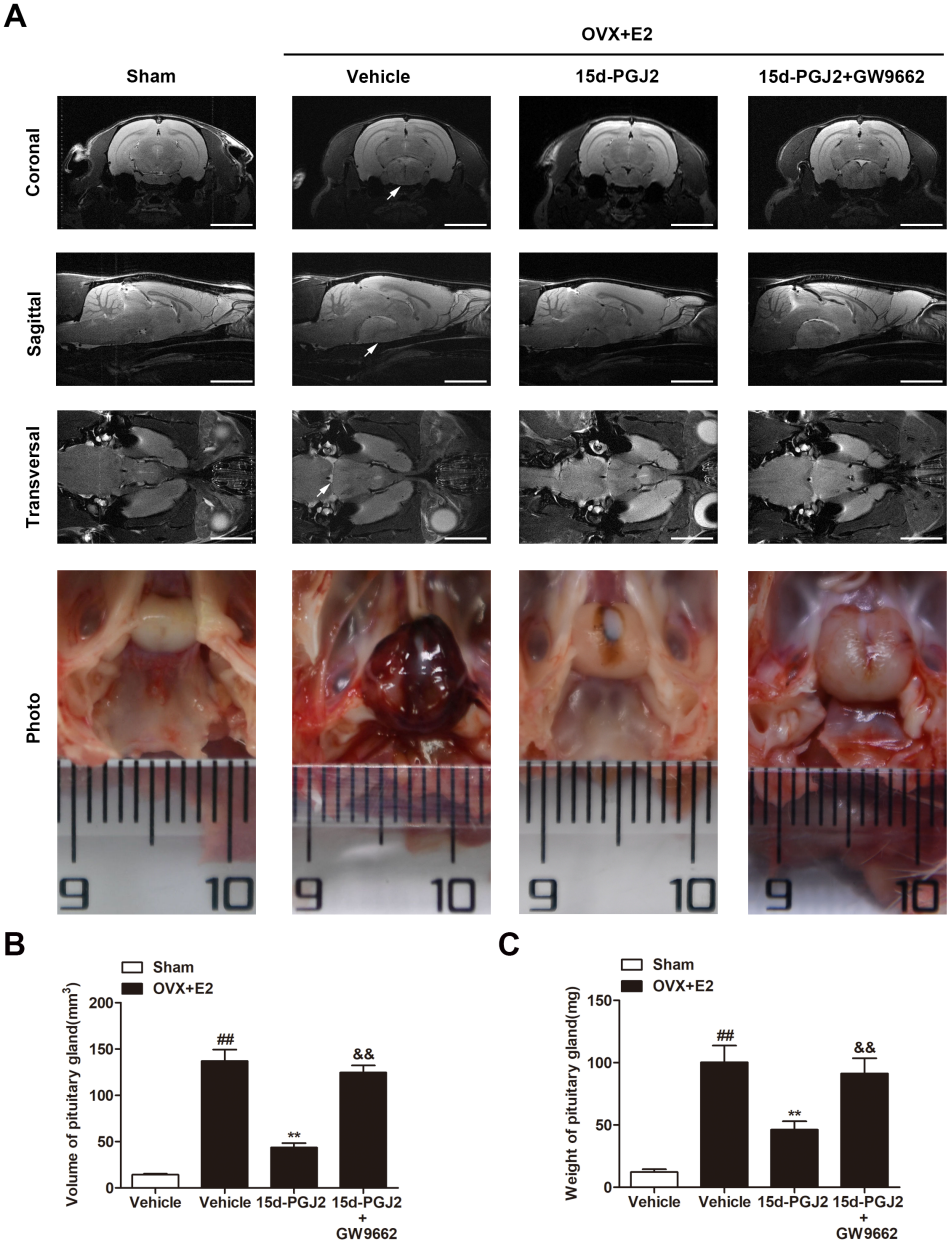
Intranasal 15d-PGJ2 reduced the tumor weight and serum PRL in E2-treated OVX rats in a PPARγ dependent manner. **(A)** MRI was used to investigate the effects of 15d-PGJ2 on the volume of the pituitary gland. Scale bar = 5 mm. Arrows indicate lactotroph PitNETs. **(B)** The volume of the pituitary gland was calculated using 3D Slicer software. **(C)** Intranasal 15d-PGJ2 decreased the weight of the pituitary gland of E2-treated OVX rats in a PPARγ-dependent manner. Data are expressed as the means ± SDs (*n* = 20 per group). Statistical analysis was performed by one-way ANOVA followed by Tukey’s *post hoc* tests. ##, *p* < 0.01 versus Sham; **, *p* < 0.01 versus vehicle-treated OVX + E2 rats; &&, *p* < 0.01 versus 15d-PGJ2-treated OVX + E2 rats.

To evaluate *in vivo* lactotroph PitNET development, pituitary MRI scanning was performed. The volume of the pituitary gland was significantly elevated in E2 (8 mg)-treated OVX rats compared with sham rats (*p* < 0.01, Figures 2A,B). Compared with vehicle treatment, intranasal 15d-PGJ2 (4 μg/100 μL/day) significantly reduced the volume and weight of the pituitary glands of E2-induced lactotroph PitNET rats (*p* < 0.01, Figures 2B,C). In contrast, cotreatment with 15d-PGJ2 (4 μg/100 μL/day) and GW9662 (1 μg/100 μL/day) abrogated the 15d-PGJ2-mediated decreases in the volume and weight of pituitary glands of E2-treated OVX rats (*p* < 0.01, Figures 2A–C).

### 3.3. Intranasal 15d-PGJ2 attenuated pathological changes and significantly decreased PRL and ERα expression in E2-treated OVX rats in a PPARγ-dependent manner

**Figure 3:**
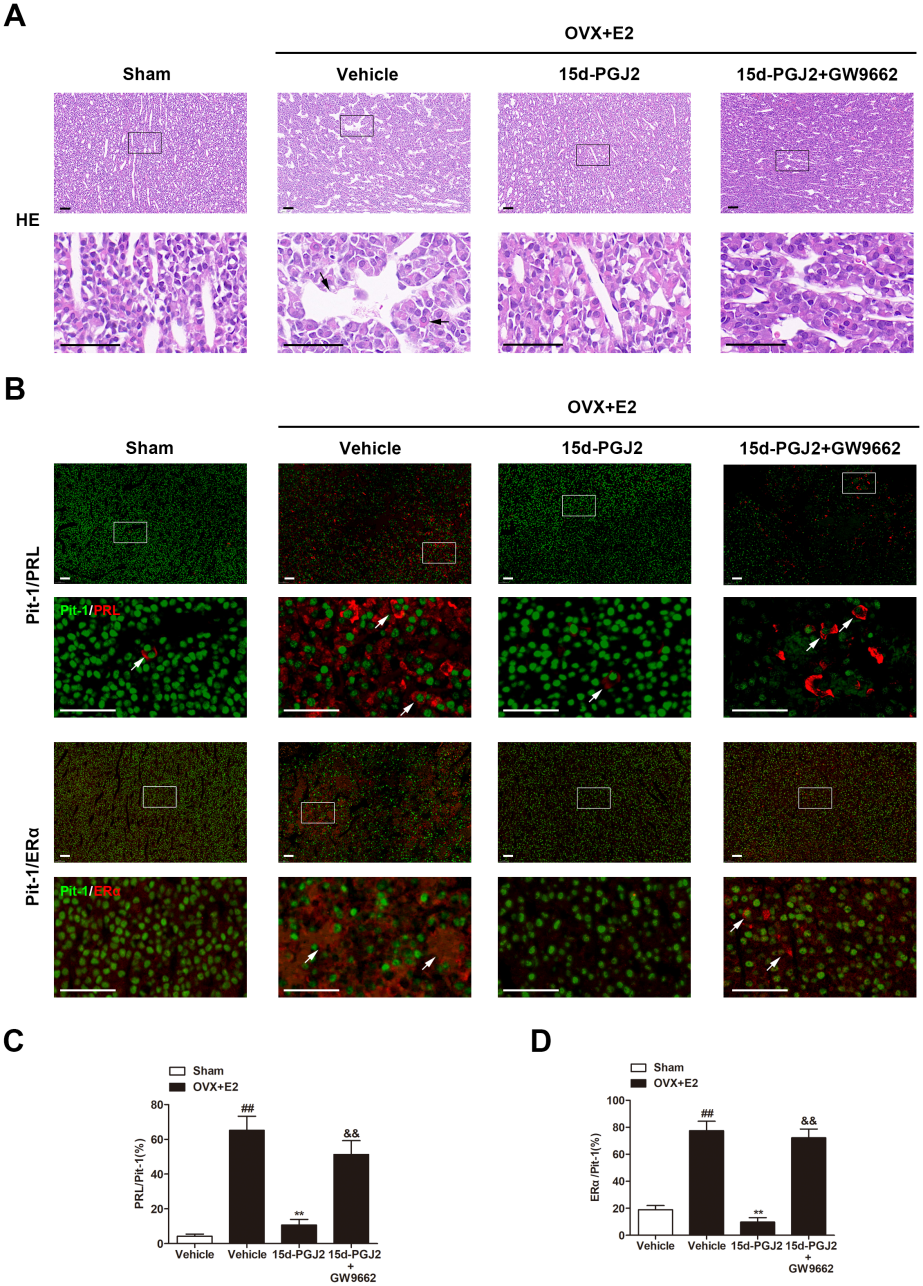
Intranasal 15d-PGJ2 attenuated pathological changes and significantly decreased PRL and ERα expression in E2-treated OVX rats in a PPARγ-dependent manner. **(A)** The pathological changes in the anterior pituitary glands of rats were detected by HE staining. The arrows indicated cellular hypertrophy and enlargement of the sinusoidal capillaries. Scale bar = 50 μm. **(B)** The pituitary sections were stained by immunofluorescence using rabbit polyclonal anti-Pit-1 antibody and mouse monoclonal anti-ERα antibody or mouse monoclonal anti-PRL antibody. Arrows indicate double immunofluorescent staining for PRL/Pit-1 or ERα/Pit-1. Scale bar = 50 μm. The proportions of PRL/Pit-1 **(C)** and ERα/Pit-1 **(D)** double immunoreactive cells were quantified. Data are expressed as the means ± SDs (*n* = 3 per group). Statistical analysis was performed by one-way ANOVA followed by Tukey’s *post hoc* tests. ##, *p* < 0.01 versus Sham; **, *p* < 0.01 versus vehicle-treated OVX + E2 rats; &&, *p* < 0.01 versus 15d-PGJ2-treated OVX + E2 rats.

The pathological changes in the anterior pituitaries of rats were detected by HE staining. As shown in Figure 3A, the anterior pituitary cells were regular in shape and size. The cytoplasm of most cells was acidophilic, and the nuclei were spherical in sham rats. However, in E2-treated OVX rats, cellular hypertrophy and enlargement of the sinusoidal capillaries were observed. Intranasal 15d-PGJ2 attenuated these pathological changes in the pituitary gland of E2-treated OVX rats. In contrast, cotreatment with 15d-PGJ2 and GW9662 blocked the effects of 15d-PGJ2.

Pit-1 plays an essential role in the transcription of PRL. ERα is an important physiological regulator of lactotroph cell proliferation. In this study, the expression of PRL, ERα and Pit-1 was detected by immunofluorescence. As shown in Figure 3B, a few cells had PRL or ERα expression in the pituitary gland of sham rats. However, the proportions of PRL/Pit-1 and ERα/Pit-1 double immunoreactive cells were increased in the pituitary gland of E2-treated OVX rats (*p* < 0.01, Figures 3B–D). 15d-PGJ2 treatment decreased the proportions of PRL/Pit-1 and ERα/Pit-1 double-positive cells in the pituitary gland of E2-treated OVX rats, and these decreases were inhibited by GW9662 cotreatment (*p* < 0.01, Figures 3B–D).

### 3.4. Intranasal 15d-PGJ2 induced apoptosis and suppressed inflammation in E2-treated OVX rats in a PPARγ-dependent manner

**Figure 4:**
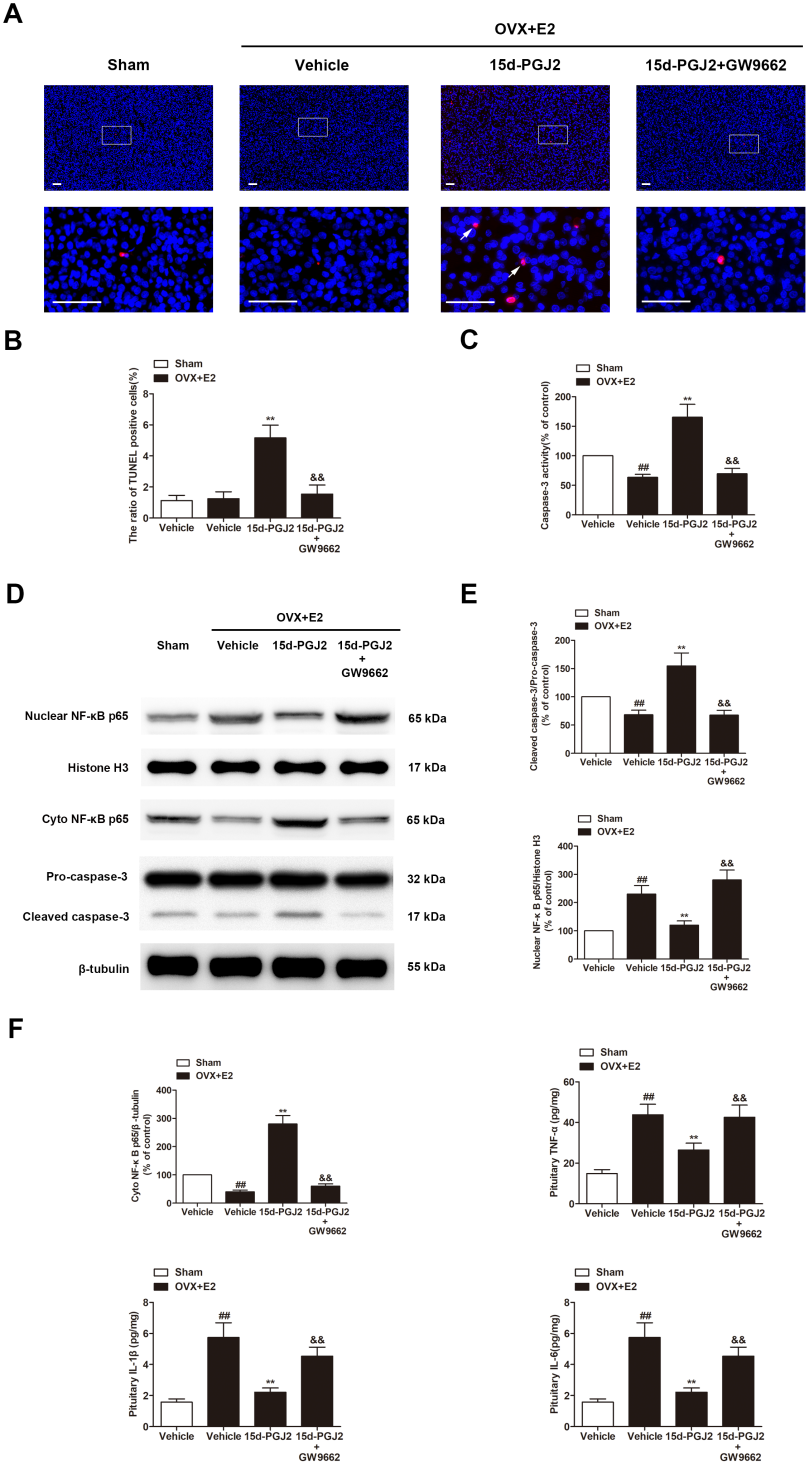
Intranasal 15d-PGJ2 induced apoptosis and suppressed inflammation in E2-treated OVX rats in a PPARγ-dependent manner. **(A)** Apoptosis in the pituitary gland was detected by TUNEL staining (*n* = 3 per group). Scale bar = 50 μm. **(B)** The ratio of TUNEL-positive cells was calculated. **(C)** Caspase-3 activity was determined by ELISA (*n* = 8 per group). **(D)** The expression of cleaved caspase-3, nuclear NF-κB p65, and cyto NF-κB p65 were determined by Western blotting (*n* = 6 per group). **(E)** Quantitative analysis of the protein expression of cleaved caspase-3 and nuclear NF-κB p65. **(F)** Quantitative analysis of the protein expression of cyto NF-κB p65. The levels of TNF-α, IL-1β, and IL-6 in the pituitary gland were detected using the respective ELISA kits (*n* = 8 per group). Data are expressed as the means ± SDs. Statistical analysis was performed by one-way ANOVA followed by Tukey’s *post hoc* tests. ##, *p* < 0.01 versus Sham; **, *p* < 0.01 versus vehicle-treated OVX + E2 rats; &&, *p* < 0.01 versus 15d-PGJ2-treated OVX + E2 rats.

Apoptosis in the pituitary gland was detected by TUNEL staining and measurement of caspase-3 expression. 15d-PGJ2 treatment dramatically increased the number of TUNEL-positive cells in the pituitary gland of E2-treated OVX rats by approximately 4-fold (*p* < 0.01, Figures 4A,B). Moreover, 15d-PGJ2 treatment significantly increased caspase-3 activity and the expression of cleaved caspase-3 in the pituitary gland of E2-treated OVX rats (*p* < 0.01, Figures 4C–E). In contrast, the 15d-PGJ2-induced increases in the numbers of TUNEL-positive cells, caspase-3 activity and the expression of cleaved caspase-3 were reversed by GW9662 cotreatment (*p* < 0.01, Figures 4A–E).

In this study, we found that the levels of inflammatory cytokines, including TNF-α, IL-1β, and IL-6, the nuclear translocation of NF-κB p65 were all significantly increased in the pituitary glands of E2-treated OVX rats compared with those in sham rats (*p* < 0.01, Figures 4D–F). However, 15d-PGJ2 treatment markedly decreased the nuclear translocation of NF-κB p65 and the levels of these inflammatory cytokines in E2-treated OVX rats in comparison to vehicle treatment (*p* < 0.01, Figures 4D–F). Cotreatment with GW9662 and 15d-PGJ2 reversed the suppression of the nuclear translocation of NF-κB p65 and inflammatory cytokine production mediated by 15d-PGJ2 (*p* < 0.01, Figures 4D–F).

### 3.5. Intranasal 15d-PGJ2 blocked autophagic flux in E2-treated OVX rats in a PPARγ-dependent manner

**Figure 5:**
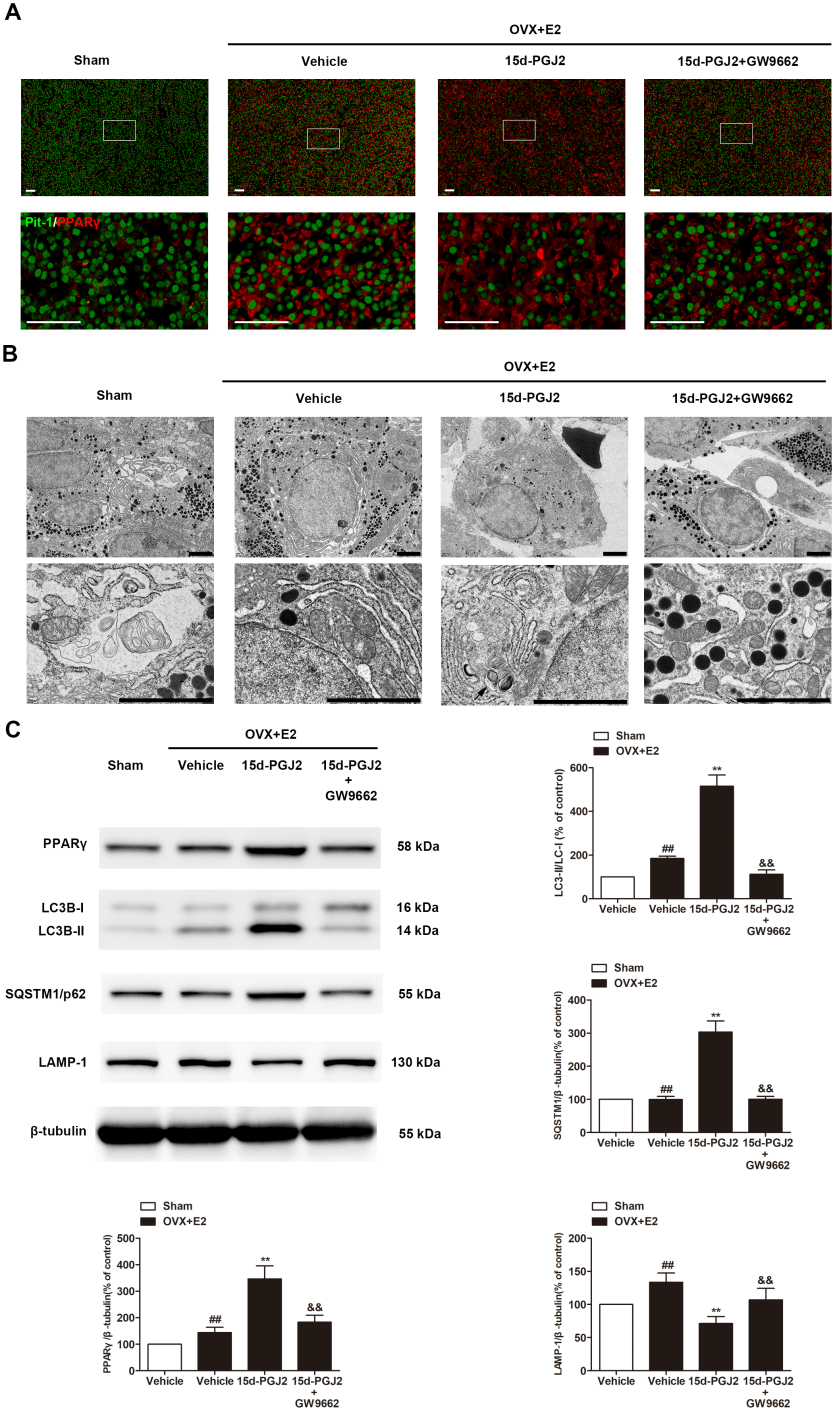
Intranasal 15d-PGJ2 blocked autophagic flux in E2-treated OVX rats in a PPARγ-dependent manner. **(A)** The expression of PPARγ in the pituitary gland was detected by immunofluorescence (*n* = 3 per group). Scale bar = 50 μm. **(B)** Ultrastructural changes in the pituitary gland were detected by TEM (*n* = 3 per group). Arrows indicate autophagosomes. Scale bar = 1 μm. **(C)** The protein expression of PPARγ, LC3, SQSTM1 and LAMP-1 was determined by Western blotting (*n* = 6 per group). Data are expressed as the means ± SDs. Statistical analysis was performed by one-way ANOVA followed by Tukey’s *post hoc* tests. ##, *p* < 0.01 versus Sham; **, *p* < 0.01 versus vehicle-treated OVX + E2 rats; &&, *p* < 0.01 versus 15d-PGJ2-treated OVX + E2 rats.

Because 15d-PGJ2 is an endogenous PPARγ agonist, the expression of PPARγ in the pituitary glands of rats was detected by immunofluorescence. As shown in Figure 5A, the expression of PPARγ was significantly increased in the pituitary glands of E2-treated OVX rats compared with those in sham rats. E2-treated OVX rats receiving 15d-PGJ2 showed a significantly higher PPARγ fluorescence intensity. In contrast, cotreatment with GW9662 and 15d-PGJ2 markedly decreased the expression of PPARγ in the pituitary glands of E2-treated OVX rats.

Ultrastructural changes in the pituitary gland were detected by TEM. As shown in Figure 5B, no typical autophagosomes were observed in the pituitary glands of sham rats. Autolysosomes were found in the pituitary glands of E2-treated OVX rats. 15d-PGJ2 treatment induced a marked accumulation of autophagosomes in the pituitary glands of E2-treated OVX rats.

Western blotting revealed that 15d-PGJ2 treatment significantly increased the protein expression of PPARγ and LC3-II. To understand the mechanism of induced LC3-II accumulation, we examined autophagic flux upon 15d-PGJ2 treatment. Compared with vehicle treatment, SQSTM1 was elevated, but the expression of LAMP-1 was downregulated in the pituitary glands of E2-treated OVX rats after 15d-PGJ2 treatment (*p* < 0.01, Figure 5C). The increase in PPARγ levels, LC3-II and SQSTM1 accumulation as well as the decrease in LAMP-1 resulting from 15d-PGJ2 treatment were all diminished by GW9662 (*p* < 0.01, Figure 5C).

## 4. Discussion

Lactotroph PitNETs are the most common pituitary tumors, but their pathogenesis is largely unknown (Neou et al., 2020). Animal models of lactotroph PitNETs provide an important resource for a better understanding of the molecular mechanisms underlying lactotroph PitNET development and the evaluation of novel therapies (Lines et al., 2016). Animal models of lactotroph PitNETs can be induced by disruption of PRL or dopamine signaling pathways or treatment with estrogen (Cao et al., 2014; Gao et al., 2017). The most common animal models of lactotroph PitNETs are Drd2^−/−^ mice, Prlr^−/−^ mice, and estrogen-treated rats. These animal models exhibit abnormal lactotroph cell proliferation and secretion and hyperprolactinaemia (Bernard et al., 2018; Liu et al., 2020). Exogenous estrogen can induce pituitary lactotroph proliferation and hyperprolactinaemia via estrogen receptors in rats (Gao et al., 2017). The susceptibility of rats to the induction of lactotroph PitNETs by estrogen treatment varies with gender, strain, and age (Lines et al., 2016). In the present study, 10 week-old female Fischer 344 rats were OVX to exhaust endogenous estrogen and subcutaneously implanted with a mini-osmotic pump containing E2. E2 treatment significantly increased the volumes and weights of lactotroph PitNETs and serum PRL in OVX rats in a dose-dependent manner. We further confirmed the animal model of lactotroph PitNETs using HE staining, immunofluorescence, and TEM. HE staining showed vascular dilatation and neoplastic nodules with large eosinophilic cells in the pituitary gland of E2-treated OVX rats. Immunofluorescence assays showed a significant elevation of PRL/Pit-1 and ERα/Pit-1 double immunoreactive cells in the pituitary gland of E2-treated OVX rats compared with sham rats. TEM showed abundant large, mature secretory granules. These results suggested that E2 treatment induced PRL-, Pit-1-, and ERα-positive lactotroph PitNETs in rats, mimicking human lactotroph PitNETs.

The pathogenesis of estrogen-induced lactotroph PitNETs involves apoptosis, autophagy, inflammation, dopaminergic synapses, estrogen signaling and PPARγ signaling (Gao et al., 2017; Liu et al., 2020). Accumulating evidence has shown that PPARγ agonists, including 15d-PGJ2 and rosiglitazone, can promote apoptosis in PitNETs (Bie et al., 2018; Zhang et al., 2021). However, the effect of the most potent endogenous PPARγ agonist, 15d-PGJ2, on lactotroph PitNETs *in vivo* and the underlying mechanisms remain largely unknown. The side effects of rosiglitazone include weight gain, fluid retention, bone loss, congestive heart failure, and a possible increased risk of myocardial infarction and bladder cancer (Bansal et al., 2020). Intranasal administration is considered an attractive route directly into the brain by circumventing the blood–brain barrier (Musumeci et al., 2019). In the present study, 15d-PGJ2 was intranasally administered to rats. We found that intranasal 15d-PGJ2 could reduce the size and weight of rat lactotroph PitNETs. 15d-PGJ2-induced the decrease in serum PRL was accompanied by a reduction of granules containing PRL. 15d-PGJ2 attenuated pathological changes and significantly decreased the proportions of PRL/Pit-1 and ERα/Pit-1 double-positive cells in the pituitary gland of rat lactotroph PitNETs. 15d-PGJ2 markedly increased the ratio of TUNEL-positive cells and the cleavage and activity of caspase-3.

The production and secretion of PRL are regulated by dopamine and estrogen, which act on the dopamine D2 receptor subtype and estrogen receptors located in lactotroph cells of the anterior pituitary gland (Bernard et al., 2019). Previous studies reported that the serum PRL levels of estrogen-induced rat lactotroph PitNETs could be decreased by treatment with dopamine D2 receptor agonists, including bromocriptine and cabergoline, and ER antagonists (Gao et al., 2017; Geng et al., 2017). The molecular mechanisms and signaling pathways involved in the development of lactotroph PitNETs and prolactin hypersecretion remain to be investigated. In the present study, intranasal 15d-PGJ2 reduced the level of serum PRL in rat lactotroph PitNETs, and this result is consistent with the decreased proportion of PRL/Pit-1 double-positive cells in the 15d-PGJ2-treated pituitary gland of rat lactotroph PitNETs. Microglial NLRP3 inflammasome activation upregulates the inflammatory cytokines IL-1β in the pituitary glands and induces lactotroph PitNETs (Wang et al., 2021). In the present study, the levels of inflammatory cytokines, including TNF-α, IL-1β, and IL-6, and the nuclear translocation of NF-κB p65 were increased in the pituitary gland of lactotroph PitNETs compared with sham rats. 15d-PGJ2 exerted anti-inflammatory properties by suppressing the nuclear translocation of NF-κB p65. Autophagy and apoptosis are two crucial cellular processes that determine cell fate and are involved in cancer (Amaravadi et al., 2019). There is crosstalk between autophagy and apoptosis (Tulipano and Giustina, 2021). PPARγ agonists have been reported to exhibit anticancer activity by inducing apoptotic or autophagic cell death pathways (Bie et al., 2018; Zhang et al., 2021). Consistent with these studies, our data showed that 15d-PGJ2 blocked autophagic flux by reducing the protein expression of the major lysosomal enzyme LAMP-1, resulting in the accumulation of SQSTM1/p62 and LC3-II. Impaired autophagy flux contributed to autophagy dysfunction because the downregulated lysosomal biogenesis upon 15d-PGJ2 treatment was not sufficient to clear the autophagosomes. Importantly, blocking PPARγ activation by GW9662 diminished 15d-PGJ2-induced autophagic cell death, which highlighted the critical role of autophagy in 15d-PGJ2-mediated tumor suppression of lactotroph PitNETs. Our experimental results provide insights into PPARγ in animal models of lactotroph PitNETs. However, the molecular mechanisms underlying how 15d-PGJ2 regulates autophagy and apoptosis remain to be investigated.

A limitation of this study is that it did not provide clinical data of patients with lactotroph PitNETs and did not examine the putative association between PPARγ expression and clinical parameters, such as tumor size, plasma prolactin levels, survival, and relapse; therefore, it would be highly relevant to study these aspects in future studies.

In conclusion, intranasal 15d-PGJ2 suppressed the growth of rat lactotroph PitNETs by inducing PPARγ-dependent apoptotic and autophagic cell death. Therefore, 15d-PGJ2 is a potential novel drug for the treatment of lactotroph PitNETs.

## Data availability statement

The original contributions presented in the study are included in the article/supplementary material, further inquiries can be directed to the corresponding authors.

## Ethics statement

The animal study was reviewed and approved by the Ethics Committee of Shenzhen Second People’s Hospital.

## Author contributions

ZL, LC, DZ, and XM wrote the main manuscript text. ZL, LC, DZ, XH, JY, WL, CW, XM, and GH prepared figures. All authors contributed to the article and approved the submitted version.

## Funding

This project was supported by the Research Fund from Shenzhen Key Laboratory of Neurosurgery (ZDSYS20140509173142601), the Shenzhen Development and Reform Commissions Stroke Screening and Prevention Public Service Platform improving program, the National Natural Science Foundation of China (81902522, 81901240, 81772685, 81760227 and 81703558), Guangdong Innovation Platform of Translational Research for Cerebrovascular Diseases, Natural Science Foundation of Guangdong Province (2018A030310647 and 2019A1515010311), the Basic research projects (subject arrangement) of Shenzhen Science and Technology Program (JCYJ20170413173149177 and JCYJ20180507184656626).

## Conflict of interest

The authors declare that the research was conducted in the absence of any commercial or financial relationships that could be construed as a potential conflict of interest.

## Publisher’s note

All claims expressed in this article are solely those of the authors and do not necessarily represent those of their affiliated organizations, or those of the publisher, the editors and the reviewers. Any product that may be evaluated in this article, or claim that may be made by its manufacturer, is not guaranteed or endorsed by the publisher.

## References

[ref1] Amaravadi R. K. Kimmelman A. C. Debnath J. (2019). Targeting autophagy in cancer: recent advances and future directions. Cancer Discov. 9, 1167–1181. doi: 10.1158/2159-8290.CD-19-0292, PMID: 31434711 PMC7306856

[ref2] Bansal G. Thanikachalam P. V. Maurya R. K. Chawla P. Ramamurthy S. (2020). An overview on medicinal perspective of thiazolidine-2,4-dione: a remarkable scaffold in the treatment of type 2 diabetes. J. Adv. Res. 23, 163–205. doi: 10.1016/j.jare.2020.01.008, PMID: 32154036 PMC7052407

[ref3] Bernard V. Villa C. Auguste A. Lamothe S. Guillou A. Martin A.. (2018). Natural and molecular history of prolactinoma: insights from a Prlr(−/−) mouse model. Oncotarget 9, 6144–6155. doi: 10.18632/oncotarget.23713, PMID: 29464061 PMC5814201

[ref4] Bernard V. Young J. Binart N. (2019). Prolactin—a pleiotropic factor in health and disease. Nat. Rev. Endocrinol. 15, 356–365. doi: 10.1038/s41574-019-0194-6, PMID: 30899100

[ref5] Bie Q. Dong H. Jin C. Zhang H. Zhang B. (2018). 15d-PGJ2 is a new hope for controlling tumor growth. Am. J. Transl. Res. 10, 648–658. PMID: 29636856 PMC5883107

[ref6] Cai W. Yang T. Liu H. Han L. Zhang K. Hu X.. (2018). Peroxisome proliferator-activated receptor γ (PPARγ): a master gatekeeper in CNS injury and repair. Prog. Neurobiol. 163–164, 27–58. doi: 10.1016/j.pneurobio.2017.10.002, PMID: 29032144 PMC6037317

[ref7] Cao L. Gao H. Gui S. Bai G. Lu R. Wang F.. (2014). Effects of the estrogen receptor antagonist fulvestrant on F344 rat prolactinoma models. J. Neuro-Oncol. 116, 523–531. doi: 10.1007/s11060-013-1351-8, PMID: 24407733

[ref8] Colin C. Meyer M. Cerella C. Kleinclauss A. Monard G. Boisbrun M.. (2018). Biotinylation enhances the anticancer effects of 15d-PGJ2 against breast cancer cells. Int. J. Oncol. 52, 1991–2000. doi: 10.3892/ijo.2018.4338, PMID: 29620161

[ref9] Gao H. Xue Y. Cao L. Liu Q. Liu C. Shan X.. (2017). ESR1 and its antagonist fulvestrant in pituitary adenomas. Mol. Cell. Endocrinol. 443, 32–41. doi: 10.1016/j.mce.2016.12.029, PMID: 28043824

[ref10] Geng X. Ma L. Li Z. Li Z. Li J. Li M.. (2017). Bromocriptine induces autophagy-dependent cell death in pituitary adenomas. World Neurosurg. 100, 407–416. doi: 10.1016/j.wneu.2017.01.052, PMID: 28137551

[ref11] Jamwal S. Blackburn J. K. Elsworth J. D. (2021). PPARγ/PGC1α signaling as a potential therapeutic target for mitochondrial biogenesis in neurodegenerative disorders. Pharmacol. Ther. 219:107705. doi: 10.1016/j.pharmthera.2020.107705, PMID: 33039420 PMC7887032

[ref12] Leng Z. G. Lin S. J. Wu Z. R. Guo Y. H. Cai L. Shang H. B.. (2017). Activation of DRD5 (dopamine receptor D5) inhibits tumor growth by autophagic cell death. Autophagy 13, 1404–1419. doi: 10.1080/15548627.2017.1328347, PMID: 28613975 PMC5584849

[ref13] Levy J. M. M. Towers C. G. Thorburn A. (2017). Targeting autophagy in cancer. Nat. Rev. Cancer 17, 528–542. doi: 10.1038/nrc.2017.53, PMID: 28751651 PMC5975367

[ref14] Li Z. Meng X. Ma G. Liu W. Li W. Cai Q.. (2022). Increasing brain glucose metabolism by ligustrazine piperazine ameliorates cognitive deficits through PPARγ-dependent enhancement of mitophagy in APP/PS1 mice. Alzheimers Res. Ther. 14:150. doi: 10.1186/s13195-022-01092-7, PMID: 36217155 PMC9552451

[ref15] Lin S. J. Wu Z. R. Cao L. Zhang Y. Leng Z. G. Guo Y. H.. (2017). Pituitary tumor suppression by combination of cabergoline and chloroquine. J. Clin. Endocrinol. Metab. 102, 3692–3703. doi: 10.1210/jc.2017-00627, PMID: 28973192

[ref16] Lines K. E. Stevenson M. Thakker R. V. (2016). Animal models of pituitary neoplasia. Mol. Cell. Endocrinol. 421, 68–81. doi: 10.1016/j.mce.2015.08.024, PMID: 26320859 PMC4721536

[ref17] Liu Y. T. Liu F. Cao L. Xue L. Gu W. T. Zheng Y. Z.. (2020). The KBTBD6/7-DRD2 axis regulates pituitary adenoma sensitivity to dopamine agonist treatment. Acta Neuropathol. 140, 377–396. doi: 10.1007/s00401-020-02180-4 32572597

[ref18] Mikulčić M. Tabrizi-Wizsy N. G. Bernhart E. M. Asslaber M. Trummer C. Windischhofer W.. (2021). 15d-PGJ(2) promotes ROS-dependent activation of MAPK-induced early apoptosis in osteosarcoma cell in vitro and in an ex Ovo CAM assay. Int. J. Mol. Sci. 22:11760. doi: 10.3390/ijms222111760, PMID: 34769194 PMC8583949

[ref19] Montaigne D. Butruille L. Staels B. (2021). PPAR control of metabolism and cardiovascular functions. Nat. Rev. Cardiol. 18, 809–823. doi: 10.1038/s41569-021-00569-6 34127848

[ref20] Musumeci T. Bonaccorso A. Puglisi G. (2019). Epilepsy disease and nose-to-brain delivery of polymeric nanoparticles: an overview. Pharmaceutics 11:118. doi: 10.3390/pharmaceutics11030118, PMID: 30871237 PMC6471219

[ref21] Neou M. Villa C. Armignacco R. Jouinot A. Raffin-Sanson M. L. Septier A.. (2020). Pangenomic classification of pituitary neuroendocrine tumors. Cancer Cell 37, 123–134.e5. doi: 10.1016/j.ccell.2019.11.002, PMID: 31883967

[ref22] Pivonello C. Patalano R. Negri M. Pirchio R. Colao A. Pivonello R.. (2021). Resistance to dopamine agonists in pituitary tumors: molecular mechanisms. Front. Endocrinol. 12:791633. doi: 10.3389/fendo.2021.791633 PMC8789681 35095761

[ref23] Soccio R. E. Chen E. R. Lazar M. A. (2014). Thiazolidinediones and the promise of insulin sensitization in type 2 diabetes. Cell Metab. 20, 573–591. doi: 10.1016/j.cmet.2014.08.005, PMID: 25242225 PMC4192012

[ref24] Srirangam Nadhamuni V. Korbonits M. (2020). Novel insights into pituitary tumorigenesis: genetic and epigenetic mechanisms. Endocr. Rev. 41, 821–846. doi: 10.1210/endrev/bnaa006, PMID: 32201880 PMC7441741

[ref25] Tae I. H. Park E. Y. Dey P. Son J. Y. Lee S. Y. Jung J. H.. (2018). Novel SIRT1 inhibitor 15-deoxy-Δ12,14-prostaglandin J2 and its derivatives exhibit anticancer activity through apoptotic or autophagic cell death pathways in SKOV3 cells. Int. J. Oncol. 53, 2518–2530. doi: 10.3892/ijo.2018.4561, PMID: 30221742 PMC6203160

[ref26] Tang C. Sun R. Wen G. Zhong C. Yang J. Zhu J.. (2019). Bromocriptine and cabergoline induce cell death in prolactinoma cells via the ERK/EGR1 and AKT/mTOR pathway respectively. Cell Death Dis. 10:335. doi: 10.1038/s41419-019-1526-0, PMID: 31000722 PMC6472389

[ref27] Tulipano G. Giustina A. (2021). Autophagy in normal pituitary and pituitary tumor cells and its potential role in the actions of somatostatin receptor ligands in acromegaly. Rev. Endocr. Metab. Disord. 22, 147–160. doi: 10.1007/s11154-021-09649-x, PMID: 33821422

[ref28] Wang X. Ma L. Ding Q. Y. Zhang W. Y. Chen Y. G. Wu J. H.. (2021). Microglial NLRP3 inflammasome activation-mediated inflammation promotes prolactinoma development. Endocr. Relat. Cancer 28, 433–448. doi: 10.1530/ERC-21-0137, PMID: 33974557

[ref29] Wasinger C. Künzl M. Minichsdorfer C. Höller C. Zellner M. Hohenegger M. (2014). Autocrine secretion of 15d-PGJ2 mediates simvastatin-induced apoptotic burst in human metastatic melanoma cells. Br. J. Pharmacol. 171, 5708–5727. doi: 10.1111/bph.12871, PMID: 25091578 PMC4290712

[ref30] Weckman A. Di Ieva A. Rotondo F. Syro L. V. Ortiz L. D. Kovacs K.. (2014). Autophagy in the endocrine glands. J. Mol. Endocrinol. 52, R151–R163. doi: 10.1530/JME-13-0241 24565917

[ref31] Yang C. B. Liu J. Tong B. C. Wang Z. Y. Zhu Z. Su C. F.. (2022). TFEB, a master regulator of autophagy and biogenesis, unexpectedly promotes apoptosis in response to the cyclopentenone prostaglandin 15d-PGJ2. Acta Pharmacol. Sin. 43, 1251–1263. doi: 10.1038/s41401-021-00711-7, PMID: 34417577 PMC9061728

[ref32] Zhang Y. Wang M. Ji C. Chen Z. Yang H. Wang L.. (2021). Treatment of acromegaly by rosiglitazone via upregulating 15-PGDH in both pituitary adenoma and liver. iScience 24:102983. doi: 10.1016/j.isci.2021.102983 34485865 PMC8403734

[ref33] Zhang F. Zhang Q. Zhu J. Yao B. Ma C. Qiao N.. (2022). Integrated proteogenomic characterization across major histological types of pituitary neuroendocrine tumors. Cell Res. 32, 1047–1067. doi: 10.1038/s41422-022-00736-5, PMID: 36307579 PMC9715725

